# The Relation of State Medicine to Architecture

**Published:** 1886-12

**Authors:** Oscar C. De Wolf

**Affiliations:** Commissioner of Health, Chicago


### The Relation of State Medicine to the Profession of Architecture. *By* Oscar C. De Wolf, A.M., M.D., *Commissioner of Health, Chicago.*

[Read before the Third Annual Convention of the Western Association of Architects, Chicago, November 19th, 1886.]

In thinking over the various definitions of state medicine, a line from an almost forgotten dramatist suggests itself to my mind: Parkes, whose *Practical Hygiene* is a classic; De Chaumont, his posthumous editor and worthy successor; Dunglison, of the *Medical Dictionary;* Mapother, Day, Southwood Smith, Chadwick, John Simon, Sir Lyon Playfair, Bowditch, Billings, Rauch and others have all tried their hands at describing and explaining a subject which still remains

Like wit, much talked of, not to be defined.*

In some respects the latest definition, that of the secretary of our State Board of Health, is the best, as it is certainly the most comprehensive.† Dr. Rauch succinctly defines *state medicine* as the connection of the state with both curative and preventive medicine for the promotion, regulation and control of measures affecting the public health. But for the object of these remarks I shall confine the term to its old signification as illustrated by Dr. Parkes, namely: The legal regulation of the conduct of individuals towards each other in strictly sanitary matters. "For example," he says, "pure air is a necessity for health; but an individual may have little control over the air which surrounds him, and which he must draw into his lungs. He may be power-

* Thomas Outway, author of "Venice Preserved," etc.

† Address in state medicine, before the American Medical Association, St. Louis, May 6th, 1886.

less to prevent other persons from contaminating his air, and thereby striking at the very foundation of his health and happiness."

What, then, shall it avail a man, though he have the wealth of Crœsus and employ the lineal descendant of Vitruvius Pollio himself to design his stately mansion, if the conditions beyond his own control necessary to ensure a prompt removal of his household-wastes, the protection of his water-supply from pollution, and the freedom of the air he breathes from contamination, be disregarded by that organization formed "to promote the general welfare," and which we call the state? Though there be observed in the building itself all the canons of all the authorities on ventilation and heating—from Dr. Arnott, who set himself the simple problem of securing "at will the temperature most congenial to the human constitution, and air as pure as blows upon the hilltop," down to the latest patentee of the newest "Automatic Zephyr Ventilator and Breath of Spring Pulsifier"; though the highest skill of the sanitary engineer and the most consummate art of the sanitary plumber be lavished upon its appointments, a man's house may, nevertheless, be anything but his castle against the foes to his health, if these be fostered and recruited by municipal neglect and state indifference.

Without the aid of State Medicine the architect will build the house in vain. He may successfully cope with the vicissitudes of a climate such as we have here in Chicago, where the mercury ranges from 22° below to 95° F. above zero—a variation of 117 degrees; where for months together less than an inch of rain may fall in 30 days, and then anon a tropical down-pour of five or six inches in 24 hours. He may even forecast the meteorological future and plan for a 25 per centum increase in the annual rainfall, such as is shown by the record to have taken place in this city within the past fifteen years. He may provide for possible seismic phenomena; construct fire-proof walls and incombustible interiors; arrange for light in quantity and direction sufficient to satisfy a German oculist, air-space and ventilation adequate to the demands of Angus Smith, plumbing scant and simple enough to meet the approval of that anonymous architect who recently announced in the *New York Evening Post* that the best plumbed house is that which contains the least plumbing. He may do all this only to find his best efforts set at naught by conditions which legalized authority alone can satisfactorily adjust or remedy.

It is this authority, directed to removing causes which injure the health of the people, that constitutes State Medicine, and it consists in that body of legislation—whether municipal ordinance, act of General Assembly or federal statute—which is intended to promote the sanitary welfare of the community. It embraces not only provisions for the prevention, exclusion or limitation of disease—by quarantine, isolation, disinfection, etc.—for enforcing vaccination, prohibiting food-adulteration, punishing the creation and maintenance of nuisances, protecting water-supplies, etc., etc., but, also, a rapidly increasing volume of legislation concerning the construction of buildings with reference to their security from fire, to their lighting, ventilation, drainage and occupancy.

"After medicine," says Mapother,* "the professions most

* Lectures on Public Health, delivered at the Royal College of Surgeons in Ireland, 1864–67.

concerned in the preservation of the public health rank those of the architect and engineer." And the genial and scholarly Irish professor of hygiene did not hesitate to acknowledge his indebtedness to "those most useful professions" when speaking of ventilation, water-supply, baths, public parks, hospitals, lodging-houses, and the dwellings of the poor. It is the fashion just now to decry at least one of these professions, and we are told in a chapter on the construction of habitations in a recent text-book of hygiene, that "Architects and builders have not kept pace with the sanitarian in the study of the conditions necessary to be observed in building a dwelling which shall answer the requirements of sanitary science."

Still more recently a well-known sanitary engineer quotes the president of the Glasgow Institute of Architects as saying: "To most architects, and especially to young architects, the construction of a building is probably, and the sanitary arrangements, shall I say certainly, the least attractive branch of their professional work; and yet it is the branch of their work which most directly affects the health and comfort of our clients." The author of "The Sanitary Drainage of Houses and Towns" ventures to think that the president of the American Institute of Architects would not differ very much from his Scotch colleague in his opinion that architects attach too little importance to their responsibilities in connection with the drainage of houses which they plan and for every detail of which they ought to have a feeling of personal accountability. And he adds for himself, based on his own experience in connection with plumbing work in houses designed and built by many of the very first architects of the country, "that the architects who know, who think or who care very much about the practical details of house-drainage are very rare; and that they are more rare among the leaders of the profession than among those who, having less artistic merit, are driven to achieve reputation in practical matters." There are architects and architects, and this sanitary engineer has evidently had to do mainly with other than members of the Western Association, among whom I happen to know many who combine both artistic merit and that sincerity which Emerson commended in him who "builded better than he knew."

It may be true that some architects are open to the strictures of these writers; but I apprehend that the true explanation of any seeming disregard of what sanitarians consider the essentials of domestic architecture lies with the *clients* of the architect in their want of definite knowledge of these essentials and of their paramount importance—rather than with the architect himself. In his work on the "Principles of Ventilation and Heating and their Practical Application," Dr. Billings touches one root of the trouble when he says: "Every one who has occasion to examine the subject discovers that it is difficult to secure good ventilation throughout a building, but very few know what the principal difficulty is. Many persons seem to suppose that it depends upon some properties of gases as yet unknown, or upon some mysteries connected with the fact that heat is a mode of motion of the molecules of matter which can only be expressed in complicated mathematical formulæ. The essential difficulty, however, which architects and engineers will find most prominent is that of cost. If the question of expense be entirely set aside, ventilation becomes a comparatively simple matter." So, too, I presume, the architect who has as tractable a client as Mr. Howells portrays in the "Rise and Fall of Silas Lapham," and who receives carte blanche as to sanitary details in general, finds no insuperable difficulty in satisfying the "requirements of sanitary science."

But even when this is withheld, it is clearly incumbent upon the architect, from his earliest interview with his client—whose sanitary ignorance may, as a rule, be safely assumed—to keep him advised of the importance of these requirements. It is the architect's duty, as Billings says, to see that "after the various additions to the plan, which will be made at the suggestion of the owner's wife, and several of his friends, on whose taste he relies, have increased the cost above what he had intended, he does not, in the spasm of economy and retrenchment which will attack him, make the reduction in the ventilation," or drainage, or other sanitary necessity, rather than on some of the ornamental work outside. What your duty is, however, I do not need to tell you; still less do I assume to instruct you in those engineering and technical details which are of the essence of your professional attainments. When the public comes to know the value of the conditions of health in the home, and is willing to pay the cost of them, the architect will not be found wanting.

> "But how can he expect that others should
> Build for him * * *
> * who for himself will take no heed at all!"

A more profitable and appropriate line of comment by a Commissioner of Public Health, accorded the privilege of addressing the members of a profession so intimately concerned with "the life o' the building," is afforded by a consideration of some of the architectural causes of disease and the rôle which state medicine may play in securing their remedy.

During the last census year the causes of a total of over 750,000 deaths were reported in detail. Of this number the group of general diseases to which diphtheria, typhoid or enteric fever, cholera infantum, etc., belong, furnished more than 200,000, and the group to which consumption belongs furnished 136,000 more. Fully one-half of the total mortality of the country is properly credited to the preventable diseases. Not all of these, it is true, are wholly preventable by any attainable perfection in the construction of habitations. Given the introduction of the particulate germs of scarlet fever, for example, into a family housed in accordance with the most approved hygienic requirements, and the disease will be as certainly developed as these germs find access into a susceptible organism. Pure air, unpolluted water, spotless cleanliness, in themselves, afford no protection against these specific communicable diseases. There is this, however, to be said concerning the whole class of eruptive fevers—small-pox, measles, scarlet fever, etc.—as well as diphtheria, whooping-cough, and other diseases whose propagation and dissemination depend upon a particular contagium, to-wit: That the subsequent safety of the dwelling into which such a disease has once obtained access, is very largely a question of its architectural construction. The solid particles of these contagia, dried up into a mere dust, absorbed by porous bodies, attached to adhesive surfaces—retain their poisonous properties for varying periods, some of them for a practically unlimited time. Before the discovery of Jenner had thrown its ægis of protection over the world, almost every dwelling was a perennial center of small-pox infection; and it is not yet definitely known how long this poison, or that of scarlet fever, diphtheria, erysipelas, hospital gangrene, etc., may remain potent in the plaster of a wall or ceiling, or in the woodwork of a room. Thanks, however, to modern sanitary science, this class of diseases is steadily losing its deadly preponderance, and out of the total of deaths each year, it now furnishes less than 10 per centum. With better housing, purer water, fresher air and more wholesome food, comes a greater resisting power to all forms of disease; and sanitary architecture goes hand-in-hand with the other branches of sanitary science in restricting the sway of preventable disease.

And yet that there still remains much to be done is shown among other facts, by the statistics of mortality from consumption and pulmonary diseases. More than 100,000 persons will have died in this country during the present year from pulmonary consumption alone—more than one-eighth of the total mortality from all causes combined. With the stock illustrations concerning the evils of impure air—the Black Hole of Calcutta, the Grotto del Cane, the bird and the bell glass, etc., we are all tolerably familiar; but, at the risk of taxing your patience, I will briefly refer to what has long seemed to me the most striking and conclusive experiment of this kind ever made. A body of men, rigidly selected by strict physical examination, at the best period of life; engaged in open-air duty of a moderate character; comfortably clothed, well fed, housed at considerable expense; and promptly cared for, even in slight illnesses, by the highest medical skill—in short, the British soldier in barracks, at home, in time of peace—was found to be dying off at the rate of 17.5 per thousand, per annum, at the same time that the mortality of both town and country populations combined was only 9.2 per thousand at the same ages, and of the country population at those ages alone, was only 7.7 per thousand.

When the cause of this great disparity came to be investigated, it was discovered that the diseases known as pulmonary were the fatal maladies, which specially affected the soldier and laid him low. It was discovered that while in civil life the deaths by pulmonary or chest diseases at the soldiers' ages were 6.3 per thousand, they amounted in the cavalry to 7.3, in the infantry of the line to 10.2, in the guards to 13.8. Of the entire number of deaths from all causes in the army, diseases of the lungs constituted the following proportion: In the cavalry 53.9 per centum, in the infantry of the line 57,277, and in the guards 67,683 per centum. Pushing their inquiries one step farther still, the reporters came at last to the kernel of their task. Why should these selected soldiers suffer so especially from diseases of the chest? Was there anything in their occupation, in their clothing, in their diet, that would account for the phenomenon and indicate the pre-disposing causes of their excessive mortality from pulmonary disease? On these points the reporters were able by the process of exclusion to remove many suspected causes. They were able to exclude night duty, want of exercise, unsuitable employment, and intemperate and debauched habits. These influences the inquirers did not, of course, ignore, but by comparison they found them insufficient to account for the disparity which was seen to exist between the soldiers and the other classes of the community.

I quote the rest of the story from the graphic pen of Benjamin Ward Richardson, whose "Field of Preventive Medicine" is at once the most charming and the most instructive volume in sanitary literature: "At last they came upon one cause which they could not exclude, and which, in accordance with the Newtonian saying, was both true and sufficient cause to account for the phenomenon. That one cause, or rather that one series of causes, was overcrowding, insufficient ventilation, and nuisances arising from latrines and defective sewerage in barracks. A single agent, *vitiated air*, acted with such intensity, especially when superadded to a certain degree of exposure, as not only to produce in the foot-guards an amount of chest-disease and especially of pulmonary consumption, greater than was produced in civil life by all the other causes united, but actually to carry off annually a number of men nearly equaling in the infantry, and actually exceeding in the guards, the number of civilians of the same age who died from all classes of disease." Dr. Richardson justly characterizes the record of these observations as the best and most forcible, because most extended and accurate, that has ever been supplied respecting the influence of confined air in the living and sleeping-apartments of men who are accustomed even to an active life and to the enjoyment of much out-door exercise. If it had been desired to carry out a great physiological experiment in order to determine how diseases of the lungs might be artificially induced in men who had been healthy up to the time of the experiment, no method could have been devised that would have led to a series of results more striking or more convincing. Neither could the experiment have been more satisfactorily concluded than was done by following the recommendations of the commissioners. They recommended that an entirely new system should be introduced into barrack life; that air, fresh and pure, should at all times circulate through the buildings, and especially through the dormitories, and that every soldier should have efficient and sufficient breathing space. Since these regulations have been in force the subjects of this experiment no longer occupy the unenviable position of being first in the ranks of those who fall victims to pulmonary consumption and other affections of the respiratory organs, but are rather the models of a lower mortality; so that as the jails, once the foci of fever, are at this time the most free of that disease, the barracks, once the foci of consumption, are now the most free of that destroying malady. In the jail in its very worst condition of foul air, the disease *typhus* was the scourge; in the barracks with foul air, but less foul, *consumption* was the scourge. With pure air substituted in both places, both diseases have been enchanted away. Well may Richardson say that lessons such as these should never be cast aside while yet in many of our best houses—best in relation to their appearance and cost, not in respect to their construction—the errors that were common in the barrack are still present, and rooms are used as sleeping-rooms which stand in the eyes of the sanitarian like so many experimental boxes for the synthetical development of pulmonary disease. "The room is too small; the room is devoid of a fireplace; the room is devoid of a ventilator; the room has a window that will open with difficulty and at best but a little way; and yet that room is used as a sleeping-room for one or it may be two persons. These are the rooms in which they who are disposed to pulmonary affection find their early fates; these rooms are the vestibules to the grave."

To this picture, as a fitting contrast, may be added the following seven points suggested as the "charter of health" which a house should have, in its construction and its architecture, to fit it for human habitation:

It must present no facilities for holding dust or the poisonous particles of disease; if it retain one it is likely to retain the other.

It must possess every facility for the removal of its impurities as fast as they are produced.

It must be free from damp.

It must be well filled with daylight from all points that can be charged with light from the sun without glare.

It must be supplied with perfectly pure air in steadily changing current.

It must be maintained at an even temperature.

It must have an abundant supply of pure water.

Such houses are by no means impossible ideals. They have already been realized in many of our suburban towns, and, by the agency of State Medicine, they may be made possible even in cities. As many of you are aware, the forthcoming session of the general assembly of this state will be urged to pass a law framed under the auspices of the Illinois State Association of Architects, to provide for the regulation and inspection of the sanitary construction and alterations or modifications of buildings in cities and villages, and to secure proper ventilation and sewerage-systems for habitable buildings, etc.

This act is intended to supplement existing legislation upon the subject, and under which in this city alone a great saving of life is annually effected.

Section 686 of the city ordinances declares that, " It shall be the duty of the commissioner-of health to enforce *all* the laws of the state and ordinances of the city in relation to the sanitary regulations of the city, and cause all nuisances to be abated with all reasonable promptness," etc.

This ordinance is very general in its character, but there are many others which apply directly to specified unsanitary conditions in all classes of buildings, and under this legislation the sanitary work of the city is divided into—

*First.* The sanitary work performed in occupied places of habitation and which includes all buildings wherein any person may dwell or lodge.

*Second.* The control of the sanitary conditions and safety as relates to egress, protecting machinery and storage of dangerous materials in places of employment or service.

*Third.* The exclusive control under the state laws of all the sanitary arrangements or conditions, such as the heating, lighting, ventilating, plumbing and drainage to be provided in every building within the city, during its construction and which is to be used as a place of habitation.

Section 1347, of the city ordinances, declares, "That no person shall hereafter erect, or cause to be erected, or converted to a new purpose by alteration, any building or structure which, or any part of which, shall be inadequate or defective in respect to ventilation, light, sewerage, or any of the usual, proper or necessary provisions or precautions for the preservation of health."

The state laws invest the commissioner-of-health with authority to control all the sanitary arrangements to be provided in any habitable building within the city.

The enforcement of these laws by the inspectors has been the means of accomplishing more valuable sanitary work than by all other ordinances combined, in that by their enforcement, all improvements made are of a *permanent character*, and place the building, except in cases of accident, in a permanently good sanitary condition, thereby benefiting all occupants uniformly throughout the city.

The construction of dark, damp, unventilated living-rooms has been wholly prohibited for the past two years. All water-closet rooms are provided with sufficient external light and ventilation, and are never permitted to be in any way directly connected with any habitable room. In fact, all sanitary conditions in the construction of buildings in this city are now enforced which are in anywise conducive to the health of the occupants.

What *state medicine* may do to regulate the conduct of individuals toward each other in strictly sanitary matters, and so to promote the end of hygiene, which aims at rendering growth more nearly perfect, decay less rapid, life more vigorous, death more remote, is well illustrated in our neighboring town of Pullman, where a corporation stands in the relation of the state to the community in this regard. I have already treated of this town from a *state medicine* standpoint, and I will only now cite its death-rate as the sufficient criterion of the success which may attend the plenary control of the sanitary conditions of human life.

The town has now been in existence six years, and its population is about 9,000—a period sufficiently long and numbers great enough to eliminate any exceptional conditions which might obtain. The death-rate of the town of Hyde Park, of which Pullman is legally and territorially a part, in which the same natural conditions exist, and which is occupied by substantially the same kind of population as that of Pullman, averages 15 per thousand annually, according to the last report of the State Board of Health. In Pullman the deaths have ranged from 6.9 to 7.6 in every thousand of population, or less than one-half the deaths in the territory immediately surrounding the town.

The average for American cities is over three times this number, and the average annual death-rate of the world is 32 out of every thousand of population. The average death-rate in the City of Mexico is 56 per thousand, or eight times the rate in Pullman. The healthful conditions here are unequaled by those in any city of the world. The lowness of the death-rate is remarkable. With one-quarter of the number of the physicians that ordinarily administer to a population of this size, Pullman has only a little more than one-quarter of the deaths usual in the same number of people.

Gentlemen of the Western Association of Architects, your profession is one of the highest exponents of the material attributes which differentiate man from the beasts which perish. Man alone makes for himself an artificial climate, outwits the elements, and makes all nature tributary in his habitation to the conveniency, strength and beauty which your ancient authority Vitruvius says are the three requisites in every structure, and without which no building can merit our esteem and approbation.

If, as Sallust says, "Every man is the architect of his own fortunes," you, gentlemen, are more than the architects of your own. To your professional intelligence, sincerity and skill, every man must trust for that without which fortune is but a dead sea fruit—a healthy life.

